# Clinical management and mortality risk in those with eating disorders and self-harm: e-cohort study using the SAIL databank

**DOI:** 10.1192/bjo.2021.23

**Published:** 2021-03-19

**Authors:** Ann John, Amanda Marchant, Joanne Demmler, Jacinta Tan, Marcos DelPozo-Banos

**Affiliations:** FFPH, Swansea University Medical School, Data Science Building, Swansea University, UK; Swansea University Medical School, Data Science Building, Swansea University, UK; Swansea University Medical School, Data Science Building, Swansea University, UK; FRCPsych, Swansea University Medical School, Data Science Building, Swansea University, UK; and Specialist Eating Disorder Team, Abertawe Bro Morgannwg University Health Board, UK; Swansea University Medical School, Data Science Building, Swansea University, UK

**Keywords:** Self-harm, eating disorders, routine data, mortality, healthcare contacts

## Abstract

**Background:**

Individuals with eating disorders who self-harm are a vulnerable group characterised by greater pathology and poorer outcomes.

**Aims:**

To explore healthcare utilisation and mortality in those with a record of: self-harm only; eating disorders only; and both co-occurring.

**Method:**

We conducted a retrospective whole population e-cohort study of individuals aged 10–64 years from 2003 to 2016. Individuals were divided into: record of self-harm only; eating disorders only; both self-harm and eating disorders; and no record of self-harm or eating disorders. We used linked routinely collected healthcare data across primary care, emergency departments, hospital admissions and out-patient appointments to examine healthcare contacts and mortality.

**Results:**

We identified 82 627 individuals: *n* = 75 165 with self-harm only; *n* = 5786 with eating disorders only; *n* = 1676 with both combined. Across all groups and settings significantly more individuals attended with significantly more contacts than the rest of the population. The combined group had the highest number of contacts per person (general practitioner, incident rate ratio IRR = 3.3, 95% CI 3.1–3.5; emergency department, IRR = 5.2, 95% CI 4.7–5.8; hospital admission, IRR = 5.2, 95% CI 4.5–6.0; out-patients, IRR = 3.9, 95% CI 3.5–4.4). Standardised mortality ratios showed the highest excess mortality overall in the self-harm only group (SMR = 3.2, 95% CI 3.1–3.3), particularly for unnatural causes of death (SMR = 17.1, 95% CI 16.3–17.9). SMRs and years of life lost showed an increased risk of mortality in younger age groups in the combined group. Adjusted hazard ratios showed increased mortality across all groups (self-harm only, HR = 5.3, 95% CI 5.2–5.5; eating disorders only, HR = 4.1, 95% CI 3.4–4.9; combined group, HR = 6.8, 95% CI 5.4–8.6).

**Conclusions:**

Individuals in all groups had higher healthcare service utilisation than the general population. The increased mortality risk in young people with a record of both eating disorders and self-harm highlights the need for early specialist intervention and enhanced support.

High levels of self-harm have been found in individuals diagnosed with eating disorders,^1,2^ where self-harm refers to any act of intentional self-injury or poisoning regardless of suicidal intent or motivation.^3,4^ The risk of self-harm is estimated at over seven times higher in those with eating disorders compared with the general population (RR = 7.5, 95% CI 7.2–7.9).^5^ A systematic review and meta-analysis found a weighted average percentage of patients with eating disorders with a history of self-harm of 27%. This review also identified a positive correlation between having an eating disorder and attempting suicide. Care setting, likely reflecting severity and complexity, has emerged as a significant predictor of self-harm, with patients in specialist eating disorder settings approximately three times more likely to report a history of self-harm compared with those recruited from general practice (GP) and community settings.^1^ Recent research has found an increased presence of other mental disorders (e.g. depression) and external causes of mortality and morbidity, including self-harm, in individuals with eating disorders.^6^

## Self-harm, eating disorders and premature mortality

People who self-harm have an increased risk of premature mortality compared with the general population, particularly from unnatural causes, i.e. unintentional injuries (accidental poisoning and other accidents) and intentional injuries (suicide and homicide).^7^ All-cause mortality following hospital presentation for self-harm was found to be more than twice that expected.^8^ Research utilising primary care data has found that young people aged 10–19 who self-harm were 9 times more likely to die by unnatural causes and 17 times more likely to die by suicide than those who did not.^7^ Eating disorders (encompassing anorexia nervosa, bulimia nervosa and eating disorder not otherwise specified (EDNOS)), although relatively rare compared with other mental disorders, are thought to be associated with one of the highest rates of mortality.^9^ Recent longitudinal research has shown a standardised mortality ratio (SMR) of 5.3 for anorexia, with lower risk for other eating disorder subtypes. Although suicide risk is elevated, leading causes of death tend to be natural causes related to eating disorder pathology.^10^

Self-harm and eating disorders share a number of epidemiological and psychosocial risk factors, such as onset in adolescence and early adulthood and associations with childhood adversity and abuse.^11,12^ Individuals with eating disorders or a history of self-harm both represent vulnerable groups with greater pathology and complex health and psychosocial needs. Both diagnoses are associated with a high risk of mortality, particularly from largely preventable causes of death, yet few studies have explored both. Epidemiological studies clarifying the pattern of service utilisation and risk of death are necessary to identify where resources and interventions can be most effectively targeted.

## Aims and objectives

The aim of this study is to examine patterns of health care utilisation, clinical management (primary, emergency department and secondary) and mortality in three mutually exclusive groups drawn from a whole population: those with a record of self-harm only; those with a record of eating disorders only; and those with a record of both self-harm and eating disorders. Interactions with demographic variables, including age, gender and deprivation, were also examined.

## Method

### Design

This is a retrospective e-cohort study.

### Data source

The SAIL databank (www.saildatabank.com) is an expanding data repository (around 3 billion records) of privacy-protected anonymised person-based linkable data, covering the population of Wales, from healthcare and public settings to support research. Robust policies, structures and controls are in place to protect privacy through a reliable matching, anonymisation and encryption process achieved in conjunction with the NHS Wales Information Service using a split file approach.^13,14^ This involves the separation of identifiable information from clinical content, identity matching and creation of anonymised linkage keys prior to reassembling and further encryption of data-sets. This is described in further detail elsewhere.^13,14^ All data within the SAIL gateway are treated in accordance with the Data Protection Act 2017 and are compliant with the General Data Protection Regulation (GDPR).

### Ethical approvals

Approval was granted by the Information Governance Review Panel (IGRP approval number 0281). This is an independent body consisting of a range of government, regulatory and professional agencies. The IGRP oversees study approvals in line with permissions already granted for the analysis of anonymous data in the SAIL databank.^13,14^

### Study population and setting

We used the following data-sets linked at an individual level: Welsh Demographic Service (WDS), a register of all individuals registered with a Welsh GP or who have ever had contact with the National Health Service (NHS); Welsh Index of Multiple Deprivation (WIMD), with all lower super output areas in Wales assigned a deprivation score derived from eight separate domains, including income, employment and education;^15^ General Practice Database (GPD), regularly updated attendance and clinical information for all GP interactions, including symptoms, diagnoses and prescriptions for 333 practices (out of 432 in Wales) covering 77% of the population; Emergency Department Data Set (EDDS), administrative and clinical information for all NHS Wales accident and emergency department attendances from August 2009 onwards; Patient Episode Database for Wales (PEDW), attendance and clinical information for all NHS Wales hospital admissions (in-patient and day cases), including data regarding diagnoses and operations performed; Outpatient Dataset (OPD), attendance information for all NHS Wales hospital out-patient appointments from 2004 onwards; Office for National Statistics (ONS) deaths register of all deaths relating to Welsh residents, including those who die outside of Wales, with information regarding date and cause of death.

A cohort of individuals living in Wales aged 10–64 years between 1 January 2003 and 30 September 2016 was created (supplementary Fig. 1, available at https://doi.org/10.1192/bjo.2021.23). Data collection began on an individual's 10th birthday or 1 January 2003, whichever was the later. Data collection ended on an individual's 65th birthday, date of death or 30 September 2016, whichever was the sooner. This cohort was further refined for the GP analysis to include only those registered with a GP contributing to SAIL. For any analysis with GP data, an individual's period of data collection began on 1 January 2003, their 10th birthday or the date of registration plus 6 months (to remove the risk of retrospective recording), whichever was the later. Data collection ended on an individual's 65th birthday, date of death or end of registration, whichever was the sooner. Each individual could contribute more than one period of data, provided that the above criteria were met. For all participants, records of self-harm or eating disorders were required to be between the above periods of data collection.

### Measures

Diagnoses were extracted from GPD records using primary care Read Codes (supplementary Appendix). The Read Codes and algorithms used to identify self-harm and eating disorders have been previously validated.^16–18^ Hospital admissions were identified on the basis of ICD-10 codes for intentional self-harm (X60–X84), with codes for self-injury or self-poisoning of undetermined intent (Y10–Y34) used to identify self-harm events, and codes F500, F501, F502, F503, F509, F982 to identify eating disorders. Dates and number of emergency department attendances and out-patient appointments were extracted. Data regarding date and cause of death were taken from the ONS deaths register. Causes of death were divided into natural and unnatural. Deaths were categorised as unnatural if the cause was recorded as self-harm, undetermined intent, accident or assault.

#### Diagnostic groups and period of follow-up

Self-harm and eating disorder events were identified using both GP and hospital admissions data. Self-harm and eating disorder events were included only if they fell within the periods of data collection described above. Participants were split into three mutually exclusive groups: self-harm only; eating disorders only; and both self-harm and eating disorders.

Participants were followed up from either the first self-harm/eating disorder date, or from study onset if no event was present, until the study end or date of death, whichever was the sooner.

#### Demographic information

Demographic information (age, gender and deprivation) was based on the midpoint of the follow-up period unless stated otherwise. Age was categorised into six groups: 10–18 years; 19–24 years; 25–34 years; 35–44 years; 45–54 years; and 55–64 years.

### Analysis

Contacts across services were examined during the period of follow-up described above for each of the diagnostic groups, using those with no record of either self-harm or eating disorders as a reference group. Percentage attending, rate and incident rate ratios (IRRs) of GP contacts, emergency department attendances, hospital admissions and out-patient appointments by diagnostic group and demographic variables were examined.

SMRs were calculated based on the overall cohort of individuals outlined above, utilising the population as whole (encompassing those both with and without a record of self-harm or eating disorders) as a reference group. Confidence intervals of the SMRs were two-tail mid-*P* exact CIs assuming Poisson distribution for the observed deaths. Additionally, years of life lost (YLL) were calculated as the difference between age at death and life expectancies for Wales (78.3 years for males, 92.3 for females, based on ONS data for Wales; www.ons.gov.uk). If an individual was older than this life expectancy on the date of death then the YLL was set to zero. The average YLL was calculated as YLL/number of deaths for each group. Cox regression was used to compute hazard ratios (HR, 95% CI) for each diagnostic group, using those with no diagnosis as the reference group. HRs were adjusted for age, gender and deprivation.

SMRs and IRRs of diagnoses, deaths and healthcare contacts were calculated using person-years at risk (PYAR) as the denominator. This is a more appropriate denominator than the number of registered cases because each individual's duration of follow-up was not fixed.^19,20^ Poisson regression was undertaken to model contacts with healthcare services as a function of diagnostic group, gender, age group and deprivation. The significance of variables in the Poisson regression modelling was assessed using Wald tests. Robust standard errors for the IRRs were used to account for clustering within GP practices. Analysis was conducted with SPSS v.22 (syntax available on request).

## Results

### Study population

Results here are reported according to the STROBE checklist. From 2003 to 2016, 2 504 108 individuals aged 10–64 years contributed 20 358 850 person-years of GP data; 82 627 individuals had a record of either self-harm or an eating disorder; 76 841 individuals had a record of self-harm (53 839 with a record in GP data and 49 434 in in-patient data; 26 432 with a record in both); 7462 individuals had a record of an eating disorder (6305 in GP data and 2003 in in-patient data; 846 with a record in both) (supplementary Fig. 1).

Individuals were categorised into three mutually exclusive groups: those with a record of self-harm with no eating disorders (*n* = 75 165); those with a record of eating disorders with no self-harm (*n* = 5786); and those with a record of both eating disorders and self-harm (*n* = 1676). Of those individuals with a record of self-harm, 2% (95% CI 2–2) also had an eating disorder record (<1% of males; 4% (95% CI 3–4) of females). Of those with a record of an eating disorder, 22% (95% CI 22–23) also had a record of self-harm (17% (95% CI 15–20) of males; 23% (95% CI 22–24) of females).

There was a clear increase in gradient of recorded self-harm in the self-harm only group with increasing deprivation (individuals with a record of self-harm per 1000 PYAR: least deprived, 1.2, 95% CI 1.1–1.2; most deprived, 3.6, 95% CI 3.6–3.6; supplementary Fig. 2). There was no change in diagnoses by deprivation quintile for the other two groups.

### Contacts with healthcare services

**Fig. 1:**
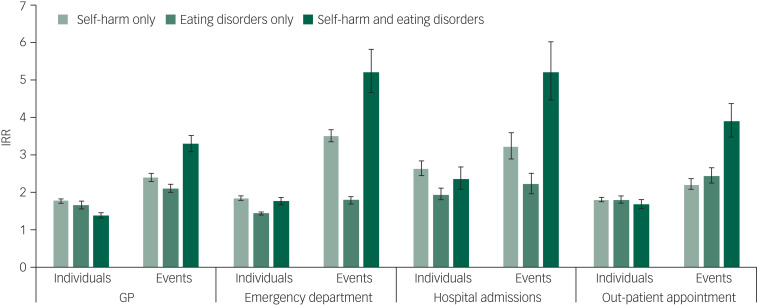
Incident rate ratios (IRRs) of contacts with healthcare services by individual, events and diagnosis. IRRs adjusted for age, gender and deprivation. Individuals refer to a count of individuals attending each service within the period of follow-up. Events refer to the number of contacts: total number of general practice (GP) appointments, emergency department attendances, hospital admissions and out-patient appointments within the follow-up period. No diagnosis was used as the reference group.

Adjusted IRRs by individuals and events across healthcare services by diagnosis are shown in Fig. 1. Individuals with no record of either eating disorders or self-harm were used as the reference group. Across all groups and healthcare services, more individuals attended with more contacts than those with no relevant diagnosis. Although the self-harm and eating disorder only groups had similar IRRs for primary care contacts, (self-harm only, IRR = 1.8, 95% CI 1.7–1.8; eating disorders only, IRR = 1.7, 95% CI 1.6–1.8), the IRR for the combined group was lower (IRR = 1.4, 95% CI 1.3–1.5). However, the combined group showed the highest IRR for the number of contacts, with more than three times as many GP events than those with no relevant diagnosis (IRR = 3.3, 95% CI 3.1–3.5). This is reflected across services, with the combined group having the highest IRRs for contacts with each service (emergency department attendances, IRR = 5.2, 95% CI 4.7–5.8; hospital admissions, IRR = 5.2, 95% CI 4.5–6.0; out-patient appointments, IRR = 3.9, 95% CI 3.5–4.4).

### Mortality

The highest unadjusted mortality rate was evident in the self-harm only group, with 10.4 (95% CI 10.1–10.7) deaths per 1000 PYAR, followed by the combined self-harm and eating disorders group, with 6.5 (95% CI 5.0–8.3) deaths per 1000 PYAR; the lowest rate was seen in the eating disorders only group, with 3.9 (95% CI 3.3–4.7) deaths per 1000 PYAR.

Crude mortality (supplementary Fig. 3) increased markedly with deprivation in the eating disorders only group (crude mortality per 1000 PYAR for the least deprived was 2.2, 95% CI 1.3–3.5; for the most deprived it was 5.3, 95% CI 3.7–7.3), less so in the self-harm only group (crude mortality per 1000 PYAR for the least deprived was 7.4, 95% CI 6.7–8.2; for the most deprived it was 8.9, 95% CI 8.4–9.3). For the combined self-harm and eating disorders group there was a less clear relationship between deprivation and mortality (crude mortality per 1000 PYAR for the least deprived was 4.5, 95% CI 2.1–8.6; for the most deprived it was 4.1, 95% CI 2.3–6.8).

#### Standardised mortality ratios

Number of deaths and SMRs for each group by age, gender and deprivation are shown in Table 1.

**Table 1:** Number of deaths, standardised mortality ratios and years of life lost (YLL) by diagnosis, gender, age, deprivation and cause of death

	Number of deaths; standardised mortality ratios (95% CI)								
	Self-harm only			Eating disorders only			Self-harm and eating disorders		
	All	Natural	Unnatural^a^	All	Natural	Unnatural	All	Natural	Unnatural
Male	2753; 3.9 (3.8–4.1)	1551; 2.4 (2.3–2.5)	1202; 18.5 (17.4–19.5)	26; 2.2 (1.4–3.1)	24; 2.2 (1.4–3.2)	<5	7; 2.2 (1.0–4.3)	<5	<5
Female	1643; 2.5 (2.4–2.7)	1124; 1.8 (1.7–1.9)	519; 18.7 (17.1–20.3)	101; 1.3 (1.1–1.6)	90; 1.2 (1.0–1.5)	11; 3.3 (1.7–5.7)	61; 2.2 (1.7–2.9)	35; 1.3 (1.0–1.9)	26; 22.3 (14.9–32.2)
Age group, years									
10–18	161; 9.0 (7.8–10.5)	37; 4.1 (2.9–5.6)	124; 13.9 (11.6–16.6)	7; 3.5 (1.6–7.0)	7; 7.0 (3.1–13.9)	0; 0 (0–0)	<5	<5	<5
19–24	290; 8.6 (7.7–9.6)	71; 4.6 (3.6–5.7)	219; 12.0 (10.5–13.7)	9; 2.8 (1.38–5.19)	7; 4.8 (2.1–9.5)	<5	12; 9.5 (5.2–16.2)	<5	7; 10.3 (4.5–20.4)
25–34	747; 12.1 (11.3–13.0)	286; 7.5 (6.7–8.4)	461; 19.7 (17.9–21.5)	20; 3.2 (2.0–4.9)	17; 4.4 (2.7–6.9)	<5	15; 8.3 (4.8–13.4)	7; 6.3 (2.8–12.4)	8; 11.7 (5.4–22.2)
35–44	1111; 8.4 (7.9–8.9)	644; 6.0 (5.5–6.5)	467; 19.0 (17.3–20.8)	23; 3.3 (2.1–4.8)	19; 3.3 (2.1–5.1)	<5	20; 7.7 (4.9–11.8)	11; 5.2 (2.8–9.1)	9; 18.7 (9.1–34.4)
45–54	1087; 4.7 (4.4–5.0)	800; 3.7 (3.5–4.0)	287; 17.3 (15.4–19.4)	43; 5.1 (3.7–6.8)	40; 5.1 (3.7–6.9)	<5	14; 4.9 (2.8–8.0)	11; 4.1 (2.2–7.2)	<5
55+	1000; 3.0 (2.8–3.2)	837; 2.6 (2.4–2.8)	163; 21.2 (18.1–24.7)	25; 2.4 (1.6–3.6)	24; 2.4 (1.6–3.5)	<5	<5	<5	0; 0 (0–0)
Deprivation quintile									
5 (least deprived)	385; 3.0 (2.7–3.3)	229; 1.9 (1.6–2.1)	156; 19.3 (16.4–22.5)	17; 0.9 (0.5–1.4)	17; 0.9 (0.6–1.5)	0; 0(0–0)	9; 1.8 (0.9–3.3)	<>; 1.3 (0.5–2.7)	<5; 9.7 (2.5–26.4)
4	530; 2.7 (2.5–2.9)	314; 1.7 (1.5–1.9)	216; 16.1 (14.0–18.3)	11; 0.5 (0.3–0.9)	<>; 0.5 (0.3–0.9)	<5	9; 1.4 (0.7–2.6)	<5	<>; 13.9 (5.7–29.0)
3	707; 2.3 (2.2–2.5)	416; 1.5 (1.3–1.6)	291; 13.7 (12.2–15.4)	20; 0.8 (0.5–1.2)	15; 0.6 (0.4–1.0)	5; 2.8 (1.0–6.2)	14; 1.7 (1.0–2.7)	6; 0.8 (0.3–1.6)	8; 13.6 (6.3–25.8)
2	993; 2.1 (2.0–2.3)	595; 1.4 (1.3–1.5)	398; 11.5 (10.4–12.7)	32; 1.1 (0.8–1.6)	<>; 1.2 (0.8–1.6)	<5	17; 1.6 (0.9–2.4)	10; 1.0 (1.0–1.8)	7; 8.7 (3.8–17.2)
1 (most deprived)	1518; 1.9 (1.8–2.0)	963; 1.3 (1.2–1.4)	555; 9.0 (8.3–9.8)	38; 1.1 (0.8–1.6)	<>; 1.1 (0.8–1.5)	<5	15; 0.9 (0.5–1.4)	10; 0.6 (0.3–1.2)	5; 3.8 (1.4–8.5)
Total	4396; 3.2 (3.1–3.3)	2675; 2.1 (2.0–2.2)	1721; 17.1 (16.3–17.9)	127; 1.2 (1.0–1.4)	114; 1.2 (1.0–1.4)	13; 1.7 (0.9–2.8)	68; 1.9 (1.5–2.4)	38; 1.1 (0.8–1.5)	30; 11.5 (7.9–16.3)
% YLL (95% CI)		51 (50–51)	49 (49–50)		88 (87–89)	12 (12–12)		49 (49–50)	51 (50–51)

<5, count of less than 5 masked for data security, and SMRs not reported because low cell counts preclude meaningful analysis; <>, number greater than 5 masked to prohibit deductive disclosure of small numbers.

a. Unnatural death is defined as death by suicide/self-harm, accident or assault.

The highest SMRs were seen in the self-harm only group, and the eating disorders only group had the lowest comparative risk of mortality, using the population as a whole as a reference group.

**Fig. 2:**
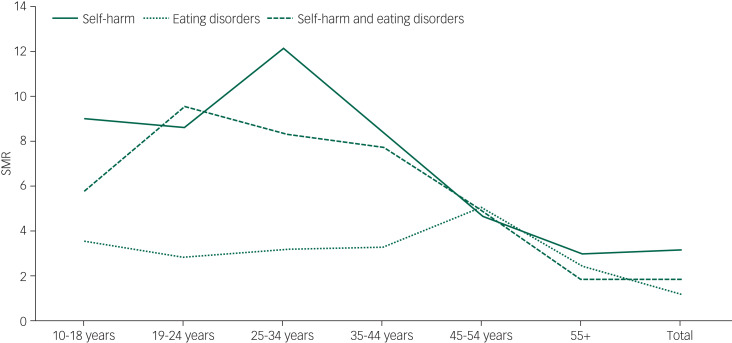
Standardised mortality ratios (SMRs) by age at midpoint of follow-up and diagnosis.

When examining all-cause mortality, higher SMRs were seen in the least deprived areas (indicating higher risk relative to others in the same deprivation fifth) across all groups, but particularly for the self-harm only group. Owing to low numbers in the eating disorders only and combined self-harm and eating disorders group it is not possible to draw meaningful conclusions about the SMRs by deprivation when breaking causes of death down into natural and unnatural. SMRs by age at midpoint of follow-up are show in Fig. 2. This shows a peak at 25–34 years for those with self-harm only, 45–54 years for eating disorders only and 19–24 years for combined self-harm and eating disorders.

SMRs also differ by cause of death. Average YLL and SMR by diagnoses and cause of death are shown in supplementary Table 1 and supplementary Fig. 4. Those in the self-harm only group and the combined self-harm and eating disorders group had particularly high SMRs for death by unnatural causes (SMR = 17.1, 95% CI 16.3–17.9; and 11.5, 95% CI 7.9–16.3) and suicide (SMR = 23.4, 95% CI 22.0–24.9; and 15.9, 95% CI 9.6–24.9). For natural causes of death, the highest SMRs were seen for the self-harm only group (SMR = 2.1, 95% CI 2.0–2.2), with similar SMRs for the eating disorders only and the combined groups (SMR = 1.2, 95% CI 1.0–1.4; and SMR = 1.1, 95% CI 0.8–1.5).

#### Average YLL and average age at death

Across causes, although the highest SMRs were seen in the self-harm only group, average YLL were consistently higher in the combined group (supplementary Table 1 and supplementary Fig. 4). This is most marked for unnatural causes of death (34.0 and 39.1 YLL respectively), particularly suicide (34.2 and 41.4 YLL respectively). For natural causes of death, the highest SMRs are seen for the self-harm only group, but the YLL are the lowest.

Table 1 shows the percentage of YLL by diagnosis and cause of death. Although SMRs in the self-harm and combined self-harm and eating disorders group are higher for unnatural causes of death, the average YLL are comparable for both natural and unnatural causes of death for these groups. For the eating disorders only group nearly 90% of YLL are for natural causes of death. This is slightly higher than in the total population. There is a lower percentage of YLL to suicide for the eating disorders only group compared with the general population (eating disorders only 3.2%, 95% CI 3.1–3.2; total population, 6.4%, 95% CI 6.3–6.4). Percentages of YLL to suicide for the self-harm only and combined self-harm and eating disorder groups were 27.2% (95% CI 26.9–30.5) and 30.2% (95% CI 29.9–30.5) respectively.

#### Survival analysis

**Fig. 3:**
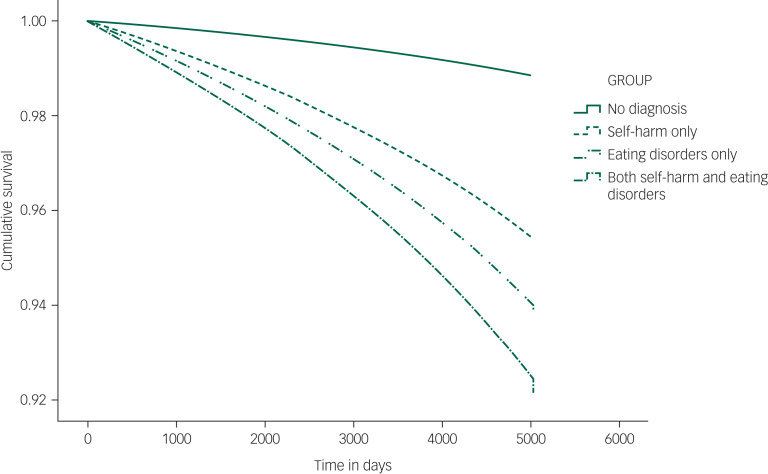
Cumulative survival by diagnostic group.

Cumulative survival by diagnostic group adjusted for age, gender and deprivation is shown in Fig. 3 (HRs are shown in supplementary Table 2). The highest adjusted HRs are seen for the combined self-harm and eating disorders group (adjusted HR = 6.8, 95% CI 5.4–8.6), followed by the self-harm only group (adjusted HR = 5.3, 95% CI 5.2–5.5), with the eating disorders only group having the lowest relative risk (adjusted HR = 4.1, 95% CI 3.4–4.9). Unadjusted HRs mirror patterns seen for SMRs.

## Discussion

To our knowledge, this study represents the first comparison of mortality and healthcare contacts between individuals with self-harm and/or eating disorders at scale in a whole population. Excess mortality has previously been demonstrated in both conditions.^6,7,10^ Although individuals with co-occurring eating disorders and self-harm have been identified as a vulnerable group,^21,22^ a large-scale analysis of all-cause mortality has not previously been conducted.

SMRs and unadjusted HRs show the highest relative risk of death for the self-harm only group, followed by the combined self-harm and eating disorders group, with the eating disorders only group having the lowest overall relative risk of death. However, HRs adjusted for age, gender and deprivation show the highest relative risk of death in the combined self-harm and eating disorders group, followed by the self-harm only group, with the lowest risk in the eating disorders only group. This is most likely due to a combination of demographic differences between the diagnostic groups. For example, in the self-harm only group just over half of individuals were female, whereas in the combined self-harm and eating disorders group, more than 90% were female. Those in the self-harm only group were more likely to come from more deprived areas than the other two groups. There are variations in relative risk of death by age discussed in more detail below.

There is a marked increase in risk of young death in the combined self-harm and eating disorders group. Although individuals in this group had a lower relative risk of death, they were more likely to die at a younger age than those in the other two groups (as demonstrated by the high SMRs for those aged 19–24 years and lower average age at death), particularly by unnatural causes of death and suicide. This suggests the need for targeted interventions for young people, particularly given the high percentage of YLL to unnatural causes in this group. Young people in more affluent areas had the lowest average age at death. More research is needed to understand why this may be the case. The role of deprivation in mortality for individuals with mental health problems is complex. Although there is generally increased excess mortality in the most deprived areas, there is some research suggesting elevated suicide risk in the most affluent areas among people with severe mental illnesses.^23^ Future research with larger sample sizes could further investigate any interactions between cause of death and deprivation.

The percentage of YLL to unnatural causes is considerably higher in the self-harm only and combined self-harm and eating disorders groups when compared with the general population. This increased risk of potentially preventable deaths demonstrates the importance of identifying self-harm and providing additional support, particularly for young people. The opposite trend is seen for those with eating disorders only, whose percentage of YLL to natural causes is slightly higher than in the population as whole. This is likely due to deaths related to physical complications of eating disorders.^10^

For the self-harm only group, higher SMRs are seen in the least deprived areas. This pattern is more pronounced for natural causes of death compared with unnatural causes of death. These SMRs compare deaths in each group with deaths in the total population within each deprivation quintile. Therefore, this does not show an increase in deaths in less deprived areas but rather a greater SMR (particularly by unnatural causes) for those in less deprived areas who have a record of self-harm compared with those who do not. A similar pattern for SMRs is seen in those with a record of both self-harm and eating disorders, again more so for unnatural causes of death, but not in the eating disorders only group.

Deprivation is a risk factor across many physical and mental health diagnoses. Diagnoses of self-harm in the most deprived fifths were found to be nearly three times those in the least deprived, in keeping with previous research.^16,24^ This is also the case for a number of other mental health conditions, including depression.^25^ The relationship between eating disorders and deprivation is less clear. Although it is commonly believed that eating disorders are more prevalent in more affluent areas, methodological problems such as small samples make this difficult to judge empirically.^26^ Some large studies have found an increase in prevalence in more affluent areas,^27^ others have found a similar distribution of eating disorder diagnoses across deprivation indices.^28^ Although the association between increased deprivation and prevalence is not seen in eating disorders the way it is for many other mental health conditions, there may be an impact of deprivation on quality of care. Lower rates of referral to specialist services following a primary care record of self-harm in more deprived compared with least deprived areas have been found in spite of a higher incidence of self-harm in these areas.^7^ Factors such as levels of referral may be having an impact on the results seen here.

### Strengths and limitations

This study is the first of its kind to link self-harm and eating disorders across primary care, emergency departments, hospital admissions, out-patients and mortality data at person level in the UK. In addition, this study utilises previously validated code lists and algorithms for the identification of eating disorders and self-harm.^16–18^ The data provide an overview of demands on services and identification of groups at risk in a way not previously possible. This study uses a large sample of individuals aged 10–64 that is representative of the rest of the UK, with levels of self-harm in eating disorders comparable to those seen in previous research.^1^ The identification of vulnerable groups could go on to inform future support and service provision.

There are some limitations to any routinely collected healthcare data. Specifically here is the lack of emergency department and out-patients data prior to 2009 and 2004 respectively, and the absence of diagnostic information in out-patient data. However, these data can still give a useful picture of demand on services and potential tailoring of support for individuals. Although the total sample size in this study was relatively large, eating disorder diagnoses are quite rare. Consequently, numbers here were not sufficient to examine finer interactions such as causes of death beyond broadly grouping into natural and unnatural/suicide. Much previous literature looking at self-harm and eating disorders divides eating disorders into subtypes.^29^ Evidence suggests that although risk of death is highest in those with anorexia nervosa, self-harm is higher in those with a diagnosis of bulimia nervosa.^29^ The decision was taken not to further divide into diagnostic subgroups here owing to the low numbers. Dividing participants into eating disorder subtypes would not have provided meaningful results and there is a recognised instability between different eating disorder diagnoses, which would render such subdivisions unhelpful.^30–32^ We note that ICD-11, released by the World Health Organization in 2018, expands the group of eating disorders to include binge eating disorder (BED) and avoidant/restrictive food intake disorder (ARFID),^33^ but this was after the data collection in this study.

The largely hidden nature of both self-harm and eating disorders makes this a challenging group to access for both research and support. Those who do not present to services or where self-harm/eating disorders are discussed but not recorded will not be captured by this analysis. This is a common feature of all research utilising routinely collected data and should be interpreted as a reflection of contacts with the healthcare system rather than a representation of the community as a whole. Community data suggest that rates of self-harm in adolescents are around 8 to 16 times those suggested by hospital studies.^34,35^ Similarly, studies have found that up to half of eating disorders may not be detected by clinical services.^36^ Future research to reach non-clinical samples in the community may allow for tailoring of support to prevent individuals from reaching crisis point.

### Implications

Findings support previous calls for self-harm assessment and self-harm-focused treatment in people with eating disorders.^1,37^ The National Confidential Inquiry into Suicide and Safety in Mental Health^38^ emphasised the need for clinical services to be equipped with the skills to address complexity associated with multiple diagnoses including eating disorders and self-harm for preventing suicides in females aged under 25, and the need for self-harm care that meets quality standards.^35^ From a clinical viewpoint, the presence of self-harm in eating disorders may indicate greater severity of pathology^21^ and risk of attempted suicide^37^ and, based on the results of this study, greater risk of mortality and healthcare utilisation. Nevertheless, the mortality rate was highest for those with self-harm only. The majority of YLL in those with a history of both self-harm and eating disorders were due to unnatural causes of death. The largely preventable nature of these deaths means that lives could be saved with appropriate support and intervention.

Although more individuals in the self-harm only group attended healthcare services, individuals in the combined self-harm and eating disorders group had a higher number of attendances per person. This may represent better engagement with services or higher utilisation due to more complex needs. It may also be a reflection of differences in help-seeking behaviour in this group. This demonstrates a higher demand on services from this group and, given their vulnerability, underscores the need for specialist services to be available.

## Data Availability

All data relevant to the study are included in the article or uploaded as online supplementary material. Person-level data are not available owing to policies and procedures in place to protect data held in the SAIL databank.

## Supplementary material

For supplementary material accompanying this paper visit http://dx.doi.org/10.1192/bjo.2021.23. click here to view supplementary material
