# Supplementary material for: Clinical management and mortality risk in those with eating disorders and self-harm: e-cohort study using the SAIL databank
Source: BJPsych Open. 2021 Mar 19;7(2):e67. doi: 10.1192/bjo.2021.23 (PMC8058850; doi:10.1192/bjo.2021.23)
Supplement: Supplementary file 1 [file bjosup.zip › S2056472421000235sup001.docx]

**Appendix A: Read codes used to identify self-harm and eating disorders in GP data**

Table 1 Read codes used to identify self-harm in GP data

| **Read Code** | **Description** |
| --- | --- |
| 14K1. | Intentional overdose of prescription only medication |
| SL... | Overdose of biological substance |
| SL90. | Antidepressant poisoning |
| SL900 | Amitriptyline poisoning |
| SL901 | Imipramine poisoning |
| SL902 | Monoamine poisoning |
| SL903 | Trazodone poisoning |
| SL90z | Anti-depressant poisoning NOS |
| TK... | Suicide and self inflicted injury |
| TK0.. | Suicide + self inflicted poisoning by solid/liquid substances |
| TK00. | Suicide + self inflicted poisoning by analgesic/antipyretic |
| TK01. | Suicide + self inflicted poisoning by barbiturates |
| TK010 | Suicide and self inflicted injury by Amylobarbitone |
| TK011 | Suicide and self inflicted injury by Barbitone |
| TK014 | Suicide and self inflicted injury by Phenobarbitone |
| TK02. | Suicide + self inflicted poisoning by oth sedatives/hypnotics |
| TK03. | Suicide + self inflicted poisoning tranquilliser/psychotropic |
| TK04. | Suicide + self inflicted poisoning by other drugs/medicines |
| TK05. | Suicide + self inflicted poisoning by drug or medicine NOS |
| TK06. | Suicide + self inflicted poisoning by agricultural chemical |
| TK07. | Suicide + self inflicted poisoning by corrosive/caustic subst |
| TK0z. | Suicide + self inflicted poisoning by solid/liquid subst NOS |
| TK1.. | Suicide + self inflicted poisoning by gases in domestic use |
| TK10. | Suicide + self inflicted poisoning by gas via pipeline |
| TK11. | Suicide + self inflicted poisoning by liquified petrol gas |
| TK1y. | Suicide and self inflicted poisoning by other utility gas |
| TK1z. | Suicide + self inflicted poisoning by domestic gases NOS |
| TK2.. | Suicide + self inflicted poisoning by other gases and vapours |
| TK20. | Suicide + self inflicted poisoning by motor veh exhaust gas |
| TK21. | Suicide and self inflicted poisoning by other carbon monoxide |
| TK2z. | Suicide + self inflicted poisoning by gases and vapours NOS |
| TK3.. | Suicide + self inflicted injury by hang/strangulate/suffocate |
| TK30. | Suicide and self inflicted injury by hanging |
| TK31. | Suicide + self inflicted injury by suffocation by plastic bag |
| TK3y. | Suicide + self inflicted inj oth mean hang/strangle/suffocate |
| TK3z. | Suicide + self inflicted inj by hang/strangle/suffocate NOS |
| TK4.. | Suicide and self inflicted injury by drowning |
| TK5.. | Suicide and self inflicted injury by firearms and explosives |
| TK51. | Suicide and self inflicted injury by shotgun |
| TK52. | Suicide and self inflicted injury by hunting rifle |
| TK54. | Suicide and self inflicted injury by other firearm |
| TK5z. | Suicide and self inflicted injury by firearms/explosives NOS |
| TK6.. | Suicide and self inflicted injury by cutting and stabbing |
| TK60. | Suicide and self inflicted injury by cutting |
| TK601 | Self inflicted lacerations to wrist |
| TK61. | Suicide and self inflicted injury by stabbing |
| TK6z. | Suicide and self inflicted injury by cutting and stabbing NOS |
| TK7.. | Suicide and self inflicted injury by jumping from high place |
| TK70. | Suicide+self inflicted injury-jump from residential premises |
| TK71. | Suicide+self inflicted injury-jump from oth manmade structure |
| TK72. | Suicide+self inflicted injury-jump from natural sites |
| TK7z. | Suicide+self inflicted injury-jump from high place NOS |
| TKx.. | Suicide and self inflicted injury by other means |
| TKx0. | Suicide + self inflicted injury-jump/lie before moving object |
| TKx00 | Suicide + self inflicted injury-jumping before moving object |
| TKx1. | Suicide and self inflicted injury by burns or fire |
| TKx2. | Suicide and self inflicted injury by scald |
| TKx3. | Suicide and self inflicted injury by extremes of cold |
| TKx4. | Suicide and self inflicted injury by electrocution |
| TKx5. | Suicide and self inflicted injury by crashing motor vehicle |
| TKx6. | Suicide and self inflicted injury by crashing of aircraft |
| TKx7. | Suicide and self inflicted injury caustic subst, excl poison |
| TKxy. | Suicide and self inflicted injury by other specified means |
| TKxz. | Suicide and self inflicted injury by other means NOS |
| TKy.. | Late effects of self inflicted injury |
| TKz.. | Suicide and self inflicted injury NOS |
| U2... | [X]Intentional self-harm |
| U20.. | [X]Intentional self poisoning by and exposure to noxious substances |
| U200. | [X]Intentional self poisoning by and exposure to nonopioid analgesics |
| U2000 | [X]Intentional self poisoning by and exposure to nonopioid analgesics, occurrence at home |
| U2001 | [X]Intentional self poisoning by and exposure to nonopioid analgesics, occurrence in residential institution |
| U2002 | [X]Intentional self poisoning by and exposure to nonopioid analgesics, occurrence at school, other institution and public administrative area |
| U2003 | [X]Intentional self poisoning by and exposure to nonopioid analgesics, occurrence at sports and athletics area |
| U2004 | [X]Intentional self poisoning by and exposure to nonopioid analgesics, occurrence on street and highway |
| U2005 | [X]Intentional self poisoning by and exposure to nonopioid analgesics, occurrence at trade and service area |
| U2006 | [X]Intentional self poisoning by and exposure to nonopioid analgesics, occurrence at industrial and construction area |
| U2007 | [X]Intentional self poisoning by and exposure to nonopioid analgesics, occurrence on farm |
| U200y | [X]Intentional self poisoning by and exposure to nonopioid analgesics, occurrence at other specified place |
| U200z | [X]Intentional self poisoning by and exposure to nonopioid analgesics, occurrence at unspecified place |
| U201. | [X]Intentional self poisoning by and exposure to antiepileptics |
| U2010 | [X]Intentional self poisoning by and exposure to antiepileptics, occurrence at home |
| U2011 | [X]Intentional self poisoning by and exposure to antiepileptics, occurrence in residential institution |
| U2012 | [X]Intentional self poisoning by and exposure to antiepileptics, occurrence at school, other institution and public administrative area |
| U2013 | [X]Intentional self poisoning by and exposure to antiepileptics, occurrence at sports and athletics area |
| U2014 | [X]Intentional self poisoning by and exposure to antiepileptics, occurrence on street and highway |
| U2015 | [X]Intentional self poisoning by and exposure to antiepileptics, occurrence at trade and service area |
| U2016 | [X]Intentional self poisoning by and exposure to antiepileptics, occurrence at industrial and construction area |
| U2017 | [X]Intentional self poisoning by and exposure to antiepileptics, occurrence on farm |
| U201y | [X]Intentional self poisoning by and exposure to antiepileptics, occurrence at other specified place |
| U201z | [X]Intentional self poisoning by and exposure to antiepileptics, occurrence at unspecified place |
| U202. | [X]Intentional self poisoning by and exposure to sedative hypnotics |
| U2020 | [X]Intentional self poisoning by and exposure to sedative hypnotics, occurrence at home |
| U2021 | [X]Intentional self poisoning by and exposure to sedative hypnotics, occurrence in residential institution |
| U2022 | [X]Intentional self poisoning by and exposure to sedative hypnotics, occurrence at school, other institution and public administrative area |
| U2023 | [X]Intentional self poisoning by and exposure to sedative hypnotics, occurrence at sports and athletics area |
| U2024 | [X]Intentional self poisoning by and exposure to sedative hypnotics, occurrence on street and highway |
| U2025 | [X]Intentional self poisoning by and exposure to sedative hypnotics, occurrence at trade and service area |
| U2026 | [X]Intentional self poisoning by and exposure to sedative hypnotics, occurrence at industrial and construction area |
| U2027 | [X]Intentional self poisoning by and exposure to sedative hypnotics, occurrence on farm |
| U202y | [X]Intentional self poisoning by and exposure to sedative hypnotics, occurrence at other specified place |
| U202z | [X]Intentional self poisoning by and exposure to sedative hypnotics, occurrence at unspecified place |
| U203. | [X]Intentional self poisoning by and exposure to antiparkinson drugs |
| U2030 | [X]Intentional self poisoning by and exposure to antiparkinson drugs, occurrence at home |
| U2031 | [X]Intentional self poisoning by and exposure to antiparkinson drugs, occurrence in residential institution |
| U2032 | [X]Intentional self poisoning by and exposure to antiparkinson drugs, occurrence at school, other institution and public administrative area |
| U2033 | [X]Intentional self poisoning by and exposure to antiparkinson drugs, occurrence at sports and athletics area |
| U2034 | [X]Intentional self poisoning by and exposure to antiparkinson drugs, occurrence on street and highway |
| U2035 | [X]Intentional self poisoning by and exposure to antiparkinson drugs, occurrence at trade and service area |
| U2036 | [X]Intentional self poisoning by and exposure to antiparkinson drugs, occurrence at industrial and construction area |
| U2037 | [X]Intentional self poisoning by and exposure to antiparkinson drugs, occurrence on farm |
| U203y | [X]Intentional self poisoning by and exposure to antiparkinson drugs, occurrence at other specified place |
| U203z | [X]Intentional self poisoning by and exposure to antiparkinson drugs, occurrence at unspecified place |
| U204. | [X]Intentional self poisoning by and exposure to psychotropic drugs |
| U2040 | [X]Intentional self poisoning by and exposure to psychotropic drugs, occurrence at home |
| U2041 | [X]Intentional self poisoning by and exposure to psychotropic drugs, occurrence in residential institution |
| U2042 | [X]Intentional self poisoning by and exposure to psychotropic drugs, occurrence at school, other institution and public administrative area |
| U2043 | [X]Intentional self poisoning by and exposure to psychotropic drugs, occurrence at sports and athletics area |
| U2044 | [X]Intentional self poisoning by and exposure to psychotropic drugs, occurrence on street and highway |
| U2045 | [X]Intentional self poisoning by and exposure to psychotropic drugs, occurrence at trade and service area |
| U2046 | [X]Intentional self poisoning by and exposure to psychotropic drugs, occurrence at industrial and construction area |
| U2047 | [X]Intentional self poisoning by and exposure to psychotropic drugs, occurrence on farm |
| U204y | [X]Intentional self poisoning by and exposure to psychotropic drugs, occurrence at other specified place |
| U204z | [X]Intentional self poisoning by and exposure to psychotropic drugs, occurrence at unspecified place |
| U205. | [X]Intentional self poisoning by and exposure to narcotic drugs |
| U2050 | [X]Intentional self poisoning by and exposure to narcotic drugs, occurrence at home |
| U2051 | [X]Intentional self poisoning by and exposure to narcotic drugs, occurrence in residential institution |
| U2052 | [X]Intentional self poisoning by and exposure to narcotic drugs, occurrence at school, other institution and public administrative area |
| U2053 | [X]Intentional self poisoning by and exposure to narcotic drugs, occurrence at sports and athletics area |
| U2054 | [X]Intentional self poisoning by and exposure to narcotic drugs, occurrence on street and highway |
| U2055 | [X]Intentional self poisoning by and exposure to narcotic drugs, occurrence at trade and service area |
| U2056 | [X]Intentional self poisoning by and exposure to narcotic drugs, occurrence at industrial and construction area |
| U2057 | [X]Intentional self poisoning by and exposure to narcotic drugs, occurrence on farm |
| U205y | [X]Intentional self poisoning by and exposure to narcotic drugs, occurrence at other specified place |
| U205z | [X]Intentional self poisoning by and exposure to narcotic drugs, occurrence at unspecified place |
| U206. | [X]Intentional self poisoning by and exposure to hallucinogens |
| U2060 | [X]Intentional self poisoning by and exposure to hallucinogens, occurrence at home |
| U2061 | [X]Intentional self poisoning by and exposure to hallucinogens, occurrence in residential institution |
| U2062 | [X]Intentional self poisoning by and exposure to hallucinogens, occurrence at school, other institution and public administrative area |
| U2063 | [X]Intentional self poisoning by and exposure to hallucinogens, occurrence at sports and athletics area |
| U2064 | [X]Intentional self poisoning by and exposure to hallucinogens, occurrence on street and highway |
| U2065 | [X]Intentional self poisoning by and exposure to hallucinogens, occurrence at trade and service area |
| U2066 | [X]Intentional self poisoning by and exposure to hallucinogens, occurrence at industrial and construction area |
| U2067 | [X]Intentional self poisoning by and exposure to hallucinogens, occurrence on farm |
| U206y | [X]Intentional self poisoning by and exposure to hallucinogens, occurrence at other specified place |
| U206z | [X]Intentional self poisoning by and exposure to hallucinogens, occurrence at unspecified place |
| U207. | [X]Intentional self poisoning by and exposure to other autonomic drugs |
| U2070 | [X]Intentional self poisoning by and exposure to other autonomic drugs, occurrence at home |
| U2071 | [X]Intentional self poisoning by and exposure to other autonomic drugs, occurrence in residential institution |
| U2072 | [X]Intentional self poisoning by and exposure to other autonomic drugs, occurrence at school, other institution and public administrative area |
| U2073 | [X]Intentional self poisoning by and exposure to other autonomic drugs, occurrence at sports and athletics area |
| U2074 | [X]Intentional self poisoning by and exposure to other autonomic drugs, occurrence on street and highway |
| U2075 | [X]Intentional self poisoning by and exposure to other autonomic drugs, occurrence at trade and service area |
| U2076 | [X]Intentional self poisoning by and exposure to other autonomic drugs, occurrence at industrial and construction area |
| U2077 | [X]Intentional self poisoning by and exposure to other autonomic drugs, occurrence on farm |
| U207y | [X]Intentional self poisoning by and exposure to other autonomic drugs, occurrence at other specified place |
| U207z | [X]Intentional self poisoning by and exposure to other autonomic drugs, occurrence at unspecified place |
| U208. | [X]Intentional self poisoning by and exposure to other and unspecified drug, medicament and biological substance |
| U2080 | [X]Intentional self poisoning by and exposure to other and unspecified drug, medicament and biological substance, occurrence at home |
| U2081 | [X]Intentional self poisoning by and exposure to other and unspecified drug, medicament and biological substance, occurrence in residential institution |
| U2082 | [X]Intentional self poisoning by and exposure to other and unspecified drug, medicament and biological substance, occurrence at school, other institution and public administrative area |
| U2083 | [X]Intentional self poisoning by and exposure to other and unspecified drug, medicament and biological substance, occurrence at sports and athletics area |
| U2084 | [X]Intentional self poisoning by and exposure to other and unspecified drug, medicament and biological substance, occurrence on street and highway |
| U2085 | [X]Intentional self poisoning by and exposure to other and unspecified drug, medicament and biological substance, occurrence at trade and service area |
| U2086 | [X]Intentional self poisoning by and exposure to other and unspecified drug, medicament and biological substance, occurrence at industrial and construction area |
| U2087 | [X]Intentional self poisoning by and exposure to other and unspecified drug, medicament and biological substance, occurrence on farm |
| U208y | [X]Intentional self poisoning by and exposure to other and unspecified drug, medicament and biological substance, occurrence at other specified place |
| U208z | [X]Intentional self poisoning by and exposure to other and unspecified drug, medicament and biological substance, occurrence at unspecified place |
| U20A. | [X]Intentional self poisoning by and exposure to organic solvents and halogenated hydrocarbons and their vapours |
| U20A0 | [X]Intentional self poisoning by and exposure to organic solvents and halogenated hydrocarbons and their vapours, occurrence at home |
| U20A1 | [X]Intentional self poisoning by and exposure to organic solvents and halogenated hydrocarbons and their vapours, occurrence in residential institution |
| U20A2 | [X]Intentional self poisoning by and exposure to organic solvents and halogenated hydrocarbons and their vapours, occurrence at school, other institution and public administrative area |
| U20A3 | [X]Intentional self poisoning by and exposure to organic solvents and halogenated hydrocarbons and their vapours, halogens, occurrence at sports and athletics area |
| U20A4 | [X]Intentional self poisoning by and exposure to organic solvents and halogenated hydrocarbons and their vapours, occurrence on street and highway |
| U20A5 | [X]Intentional self poisoning by and exposure to organic solvents and halogenated hydrocarbons and their vapours, occurrence at trade and service area |
| U20A6 | [X]Intentional self poisoning by and exposure to organic solvents and halogenated hydrocarbons and their vapours, occurrence at industrial and construction area |
| U20A7 | [X]Intentional self poisoning by and exposure to organic solvents and halogenated hydrocarbons and their vapours, occurrence on farm |
| U20Ay | [X]Intentional self poisoning by and exposure to organic solvents and halogenated hydrocarbons and their vapours, occurrence at other specified place |
| U20Az | [X]Intentional self poisoning by and exposure to organic solvents and halogenated hydrocarbons and their vapours, halogens, occurrence at unspecified place |
| U20B. | [X]Intentional self poisoning by and exposure to other gas and vapours |
| U20B0 | [X]Intentional self poisoning by and exposure to other gas and vapours, occurrence at home |
| U20B1 | [X]Intentional self poisoning by and exposure to other gas and vapours, occurrence in residential institution |
| U20B2 | [X]Intentional self poisoning by and exposure to other gas and vapour, occurrence at school, other institution and public administrative area |
| U20B3 | [X]Intentional self poisoning by and exposure to other gas and vapour, occurrence at sports and athletics area |
| U20B4 | [X]Intentional self poisoning by and exposure to other gas and vapour, occurrence on street and highway |
| U20B5 | [X]Intentional self poisoning by and exposure to other gas and vapour, occurrence at trade and service area |
| U20B6 | [X]Intentional self poisoning by and exposure to other gas and vapour, occurrence at industrial and construction area |
| U20B7 | [X]Intentional self poisoning by and exposure to other gas and vapour, occurrence on farm |
| U20By | [X]Intentional self poisoning by and exposure to other gas and vapour, occurrence at other specified place |
| U20Bz | [X]Intentional self poisoning by and exposure to other gas and vapour, occurrence at unspecified place |
| U20C. | [X]Intentional self poisoning by and exposure to pesticides |
| U20C0 | [X]Intentional self poisoning by and exposure to pesticides, occurrence at home |
| U20C1 | [X]Intentional self poisoning by and exposure to pesticides, occurrence in residential institution |
| U20C2 | [X]Intentional self poisoning by and exposure to pesticides, occurrence at school, other institution and public administrative area |
| U20C3 | [X]Intentional self poisoning by and exposure to pesticides, occurrence at sports and athletics area |
| U20C4 | [X]Intentional self poisoning by and exposure to pesticides, occurrence on street and highway |
| U20C5 | [X]Intentional self poisoning by and exposure to pesticides, occurrence at trade and service area |
| U20C6 | [X]Intentional self poisoning by and exposure to pesticides, occurrence at industrial and construction area |
| U20C7 | [X]Intentional self poisoning by and exposure to pesticides, occurrence on farm |
| U20Cy | [X]Intentional self poisoning by and exposure to pesticides, occurrence at other specified place |
| U20Cz | [X]Intentional self poisoning by and exposure to pesticides, occurrence at unspecified place |
| U20y. | [X]Intentional self poisoning by and exposure to other and unspecified chemicals and noxious substances |
| U20y0 | [X]Intentional self poisoning by and exposure to other and unspecified chemicals and noxious substances, occurrence at home |
| U20y1 | [X]Intentional self poisoning by and exposure to other and unspecified chemicals and noxious substances, occurrence in residential institution |
| U20y2 | [X]Intentional self poisoning by and exposure to other and unspecified chemicals and noxious substances, occurrence at school, other institution and public administrative area |
| U20y3 | [X]Intentional self poisoning by and exposure to other and unspecified chemicals and noxious substances, occurrence at sports and athletics area |
| U20y4 | [X]Intentional self poisoning by and exposure to other and unspecified chemicals and noxious substances, occurrence on street and highway |
| U20y5 | [X]Intentional self poisoning by and exposure to other and unspecified chemicals and noxious substances, occurrence at trade and service area |
| U20y6 | [X]Intentional self poisoning by and exposure to other and unspecified chemicals and noxious substances, occurrence at industrial and construction area |
| U20y7 | [X]Intentional self poisoning by and exposure to other and unspecified chemicals and noxious substances, occurrence on farm |
| U20yy | [X]Intentional self poisoning by and exposure to other and unspecified chemicals and noxious substances, occurrence at other specified place |
| U20yz | [X]Intentional self poisoning by and exposure to other and unspecified chemicals and noxious substances, occurrence at unspecified place |
| U21.. | [X]Intentional self harm by hanging, strangulation and suffocation |
| U210. | [X]Intentional self harm by hanging, strangulation and suffocation, occurrence at home |
| U211. | [X]Intentional self harm by hanging, strangulation and suffocation, occurrence in residential institution |
| U212. | [X]Intentional self harm by hanging, strangulation and suffocation, occurrence at school, other institution and public administrative area |
| U213. | [X]Intentional self harm by hanging, strangulation and suffocation, occurrence at sports and athletics area |
| U214. | [X]Intentional self harm by hanging, strangulation and suffocation, occurrence on street and highway |
| U215. | [X]Intentional self harm by hanging, strangulation and suffocation, occurrence at trade and service area |
| U216. | [X]Intentional self harm by hanging, strangulation and suffocation, occurrence at industrial and construction area |
| U217. | [X]Intentional self harm by hanging, strangulation and suffocation, occurrence on farm |
| U21y. | [X]Intentional self harm by hanging, strangulation and suffocation, occurrence at other specified place |
| U21z. | [X]Intentional self harm by hanging, strangulation and suffocation, occurrence at unspecified place |
| U22.. | [X]Intentional self harm by drowning and submersion |
| U220. | [X]Intentional self harm by drowning and submersion, occurrence at home |
| U221. | [X]Intentional self harm by drowning and submersion, occurrence in residential institution |
| U222. | [X]Intentional self harm by drowning and submersion, occurrence at school, other institution and public administrative area |
| U223. | [X]Intentional self harm by drowning and submersion, occurrence at sports and athletics area |
| U224. | [X]Intentional self harm by drowning and submersion, occurrence on street and highway |
| U225. | [X]Intentional self harm by drowning and submersion, occurrence at trade and service area |
| U226. | [X]Intentional self harm by drowning and submersion, occurrence at industrial and construction area |
| U227. | [X]Intentional self harm by drowning and submersion, occurrence on farm |
| U22y. | [X]Intentional self harm by drowning and submersion, occurrence at other specified place |
| U22z. | [X]Intentional self harm by drowning and submersion, occurrence at unspecified place |
| U23.. | [X]Intentional self harm by handgun discharge |
| U230. | [X]Intentional self harm by handgun discharge, occurrence at home |
| U231. | [X]Intentional self harm by handgun discharge, occurrence in residential institution |
| U232. | [X]Intentional self harm by handgun discharge, occurrence at school, other institution and public administrative area |
| U233. | [X]Intentional self harm by handgun discharge, occurrence at sports and athletics area |
| U234. | [X]Intentional self harm by handgun discharge, occurrence on street and highway |
| U235. | [X]Intentional self harm by handgun discharge, occurrence at trade and service area |
| U236. | [X]Intentional self harm by handgun discharge, occurrence at industrial and construction area |
| U237. | [X]Intentional self harm by handgun discharge, occurrence on farm |
| U23y. | [X]Intentional self harm by handgun discharge, occurrence at other specified place |
| U23z. | [X]Intentional self harm by handgun discharge, occurrence at unspecified place |
| U24.. | [X]Intentional self harm by rifle, shotgun and larger firearm discharge |
| U240. | [X]Intentional self harm by rifle, shotgun and larger firearm discharge, occurrence at home |
| U241. | [X]Intentional self harm by rifle, shotgun and larger firearm discharge, occurrence in residential institution |
| U242. | [X]Intentional self harm by rifle, shotgun and larger firearm discharge, occurrence at school, other institution and public administrative area |
| U243. | [X]Intentional self harm by rifle, shotgun and larger firearm discharge, occurrence at sports and athletics area |
| U244. | [X]Intentional self harm by rifle, shotgun and larger firearm discharge, occurrence on street and highway |
| U245. | [X]Intentional self harm by rifle, shotgun and larger firearm discharge, occurrence at trade and service area |
| U246. | [X]Intentional self harm by rifle, shotgun and larger firearm discharge, occurrence at industrial and construction area |
| U247. | [X]Intentional self harm by rifle, shotgun and larger firearm discharge, occurrence on farm |
| U24y. | [X]Intentional self harm by rifle, shotgun and larger firearm discharge, occurrence at other specified place |
| U24z. | [X]Intentional self harm by rifle, shotgun and larger firearm discharge, occurrence at unspecified place |
| U25.. | [X]Intentional self harm by other and unspecified firearm discharge |
| U250. | [X]Intentional self harm by other and unspecified firearm discharge, occurrence at home |
| U251. | [X]Intentional self harm by other and unspecified firearm discharge, occurrence in residential institution |
| U252. | [X]Intentional self harm by other and unspecified firearm discharge, occurrence at school, other institution and public administrative area |
| U253. | [X]Intentional self harm by other and unspecified firearm discharge, occurrence at sports and athletics area |
| U254. | [X]Intentional self harm by other and unspecified firearm discharge, occurrence on street and highway |
| U255. | [X]Intentional self harm by other and unspecified firearm discharge, occurrence at trade and service area |
| U256. | [X]Intentional self harm by other and unspecified firearm discharge, occurrence at industrial and construction area |
| U257. | [X]Intentional self harm by other and unspecified firearm discharge, occurrence on farm |
| U25y. | [X]Intentional self harm by other and unspecified firearm discharge, occurrence at other specified place |
| U25z. | [X]Intentional self harm by other and unspecified firearm discharge, occurrence at unspecified place |
| U26.. | [X]Intentional self harm by explosive material |
| U260. | [X]Intentional self harm by explosive material, occurrence at home |
| U261. | [X]Intentional self harm by explosive material, occurrence in residential institution |
| U262. | [X]Intentional self harm by explosive material, occurrence at school, other institution and public administrative area |
| U263. | [X]Intentional self harm by explosive material, occurrence at sports and athletics area |
| U264. | [X]Intentional self harm by explosive material, occurrence on street and highway |
| U265. | [X]Intentional self harm by explosive material, occurrence at trade and service area |
| U266. | [X]Intentional self harm by explosive material, occurrence at industrial and construction area |
| U267. | [X]Intentional self harm by explosive material, occurrence on farm |
| U26y. | [X]Intentional self harm by explosive material, occurrence at other specified place |
| U26z. | [X]Intentional self harm by explosive material, occurrence at unspecified place |
| U27.. | [X]Intentional self harm by smoke, fire and flames |
| U270. | [X]Intentional self harm by smoke, fire and flames, occurrence at home |
| U271. | [X]Intentional self harm by smoke, fire and flames, occurrence in residential institution |
| U272. | [X]Intentional self harm by smoke, fire and flames, occurrence at school, other institution and public administrative area |
| U273. | [X]Intentional self harm by smoke, fire and flames, occurrence at sports and athletics area |
| U274. | [X]Intentional self harm by smoke, fire and flames, occurrence on street and highway |
| U275. | [X]Intentional self harm by smoke, fire and flames, occurrence at trade and service area |
| U276. | [X]Intentional self harm by smoke, fire and flames, occurrence at industrial and construction area |
| U277. | [X]Intentional self harm by smoke, fire and flames, occurrence on farm |
| U27y. | [X]Intentional self harm by smoke, fire and flames, occurrence at other specified place |
| U27z. | [X]Intentional self harm by smoke, fire and flames, occurrence at unspecified place |
| U28.. | [X]Intentional self harm by steam, hot vapours and hot objects |
| U280. | [X]Intentional self harm by steam, hot vapours and hot objects, occurrence at home |
| U281. | [X]Intentional self harm by steam, hot vapours and hot objects, occurrence in residential institution |
| U282. | [X]Intentional self harm by steam, hot vapours and hot objects, occurrence at school, other institution and public administrative area |
| U283. | [X]Intentional self harm by steam, hot vapours and hot objects, occurrence at sports and athletics area |
| U284. | [X]Intentional self harm by steam, hot vapours and hot objects, occurrence on street and highway |
| U285. | [X]Intentional self harm by steam, hot vapours and hot objects, occurrence at trade and service area |
| U286. | [X]Intentional self harm by steam, hot vapours and hot objects, occurrence at industrial and construction area |
| U287. | [X]Intentional self harm by steam, hot vapours and hot objects, occurrence on farm |
| U28y. | [X]Intentional self harm by steam, hot vapours and hot objects, occurrence at other specified place |
| U28z. | [X]Intentional self harm by steam, hot vapours and hot objects, occurrence at unspecified place |
| U29.. | [X]Intentional self harm by sharp object |
| U290. | [X]Intentional self harm by sharp object, occurrence at home |
| U291. | [X]Intentional self harm by sharp object, occurrence in residential institution |
| U292. | [X]Intentional self harm by sharp object, occurrence at school, other institution and public administrative area |
| U293. | [X]Intentional self harm by sharp object, occurrence at sports and athletics area |
| U294. | [X]Intentional self harm by sharp object, occurrence on street and highway |
| U295. | [X]Intentional self harm by sharp object, occurrence at trade and service area |
| U296. | [X]Intentional self harm by sharp object, occurrence at industrial and construction area |
| U297. | [X]Intentional self harm by sharp object, occurrence on farm |
| U29y. | [X]Intentional self harm by sharp object, occurrence at other specified place |
| U29z. | [X]Intentional self harm by sharp object, occurrence at unspecified place |
| U2A.. | [X]Intentional self harm by blunt object |
| U2A0. | [X]Intentional self harm by blunt object, occurrence at home |
| U2A1. | [X]Intentional self harm by blunt object, occurrence in residential institution |
| U2A2. | [X]Intentional self harm by blunt object, occurrence at school, other institution and public administrative area |
| U2A3. | [X]Intentional self harm by blunt object, occurrence at sports and athletics area |
| U2A4. | [X]Intentional self harm by blunt object, occurrence on street and highway |
| U2A5. | [X]Intentional self harm by blunt object, occurrence at trade and service area |
| U2A6. | [X]Intentional self harm by blunt object, occurrence at industrial and construction area |
| U2A7. | [X]Intentional self harm by blunt object, occurrence on farm |
| U2Ay. | [X]Intentional self harm by blunt object, occurrence at other specified place |
| U2Az. | [X]Intentional self harm by blunt object, occurrence at unspecified place |
| U2B.. | [X]Intentional self harm by jumping from a high place |
| U2B0. | [X]Intentional self harm by jumping from a high place, occurrence at home |
| U2B1. | [X]Intentional self harm by jumping from a high place, occurrence in residential institution |
| U2B2. | [X]Intentional self harm by jumping from a high place, occurrence at school, other institution and public administrative area |
| U2B3. | [X]Intentional self harm by jumping from a high place, occurrence at sports and athletics area |
| U2B4. | [X]Intentional self harm by jumping from a high place, occurrence on street and highway |
| U2B5. | [X]Intentional self harm by jumping from a high place, occurrence at trade and service area |
| U2B6. | [X]Intentional self harm by jumping from a high place, occurrence at industrial and construction area |
| U2B7. | [X]Intentional self harm by jumping from a high place, occurrence on farm |
| U2By. | [X]Intentional self harm by jumping from a high place, occurrence at other specified place |
| U2Bz. | [X]Intentional self harm by jumping from a high place, occurrence at unspecified place |
| U2C.. | [X]Intentional self harm by jumping or lying before moving object |
| U2C0. | [X]Intentional self harm by jumping or lying before moving object, occurrence at home |
| U2C1. | [X]Intentional self harm by jumping or lying before moving object, occurrence in residential institution |
| U2C2. | [X]Intentional self harm by jumping or lying before moving object, occurrence at school, other institution and public administrative area |
| U2C3. | [X]Intentional self harm by jumping or lying before moving object, occurrence at sports and athletics area |
| U2C4. | [X]Intentional self harm by jumping or lying before moving object, occurrence on street and highway |
| U2C5. | [X]Intentional self harm by jumping or lying before moving object, occurrence at trade and service area |
| U2C6. | [X]Intentional self harm by jumping or lying before moving object, occurrence at industrial and construction area |
| U2C7. | [X]Intentional self harm by jumping or lying before moving object, occurrence on farm |
| U2Cy. | [X]Intentional self harm by jumping or lying before moving object, occurrence at other specified place |
| U2Cz. | [X]Intentional self harm by jumping or lying before moving object, occurrence at unspecified place |
| U2D.. | [X]Intentional self harm by crashing of motor vehicle |
| U2D0. | [X]Intentional self harm by crashing of motor vehicle, occurrence at home |
| U2D1. | [X]Intentional self harm by crashing of motor vehicle, occurrence in residential institution |
| U2D2. | [X]Intentional self harm by crashing of motor vehicle, occurrence at school, other institution and public administrative area |
| U2D3. | [X]Intentional self harm by crashing of motor vehicle, occurrence at sports and athletics area |
| U2D4. | [X]Intentional self harm by crashing of motor vehicle, occurrence on street and highway |
| U2D5. | [X]Intentional self harm by crashing of motor vehicle, occurrence at trade and service area |
| U2D6. | [X]Intentional self harm by crashing of motor vehicle, occurrence at industrial and construction area |
| U2D7. | [X]Intentional self harm by crashing of motor vehicle, occurrence on farm |
| U2Dy. | [X]Intentional self harm by crashing of motor vehicle, occurrence at other specified place |
| U2Dz. | [X]Intentional self harm by crashing of motor vehicle, occurrence at unspecified place |
| U2E.. | [X]Self mutilation |
| U2y.. | [X]Intentional self harm by other specified means |
| U2y0. | [X]Intentional self harm by other specified means, occurrence at home |
| U2y1. | [X]Intentional self harm by other specified means, occurrence in residential institution |
| U2y2. | [X]Intentional self harm by other specified means, occurrence at school, other institution and public administrative area |
| U2y3. | [X]Intentional self harm by other specified means, occurrence at sports and athletics area |
| U2y4. | [X]Intentional self harm by other specified means, occurrence on street and highway |
| U2y5. | [X]Intentional self harm by other specified means, occurrence at trade and service area |
| U2y6. | [X]Intentional self harm by other specified means, occurrence at industrial and construction area |
| U2y7. | [X]Intentional self harm by other specified means, occurrence on farm |
| U2yy. | [X]Intentional self harm by other specified means, occurrence at other specified place |
| U2yz. | [X]Intentional self harm by other specified means, occurrence at unspecified place |
| U2z.. | [X]Intentional self harm by unspecified means |
| U2z0. | [X]Intentional self harm by unspecified means, occurrence at home |
| U2z1. | [X]Intentional self harm by unspecified means, occurrence in residential institution |
| U2z2. | [X]Intentional self harm by unspecified means, occurrence at school, other institution and public administrative area |
| U2z3. | [X]Intentional self harm by unspecified means, occurrence at sports and athletics area |
| U2z4. | [X]Intentional self harm by unspecified means, occurrence on street and highway |
| U2z5. | [X]Intentional self harm by unspecified means, occurrence at trade and service area |
| U2z6. | [X]Intentional self harm by unspecified means, occurrence at industrial and construction area |
| U2z7. | [X]Intentional self harm by unspecified means, occurrence on farm |
| U2zy. | [X]Intentional self harm by unspecified means, occurrence at other specified place |
| U2zz. | [X]Intentional self harm by unspecified means, occurrence at unspecified place |
| U41.. | [X]Hanging strangulation + suffocation undetermined intent |
| U44.. | [X]Rifle shotgun+larger firearm discharge undetermin intent |
| U45.. | [X]Other+unspecified firearm discharge undetermined intent |
| U4B.. | [X]Falling jumping/pushed from high place undeterm intent |
| U4Bz. | [X]Fall jump/push frm high plce undt intnt occ unspecif plce |
| U72.. | [X]Sequel intentn self-harm assault+event of undeterm intent |
| U720. | [X]Sequelae of intentional self-harm |
| ZRLfC12 | Health of the Nation Outcome Scales item 2 – nonaccidental self-injury |
| ZX... | Self-harm |
| ZX1.. | Self-injurious behaviour |
| ZX11. | Biting self |
| ZX12. | Burning self |
| ZX13. | Cutting self |
| ZX15. | Drowning self |
| ZX18. | Hanging self |
| ZX19. | Hitting self |
| ZX191 | Punching self |
| ZX192 | Slapping self |
| ZX1B. | Jumping from height |
| ZX1B1 | Jumping from building |
| ZX1B2 | Jumping from bridge |
| ZX1B3 | Jumping from cliff |
| ZX1C. | Nipping self |
| ZX1E. | Pinching self |
| ZX1G. | Scratches self |
| ZX1H. | Self-asphyxiation |
| ZX1H1 | Self-strangulation |
| ZX1H2 | Self-suffocation |
| ZX1I. | Self-scalding |
| ZX1J. | Self-electrocution |
| ZX1K. | Self-incineration |
| ZX1L. | Self-mutilation |
| ZX1L1 | Self-mutilation of hands |
| ZX1L2 | Self-mutilation of genitalia |
| ZX1L3 | Self-mutilation of penis |
| ZX1L6 | Self-mutilation of ears |
| ZX1LD | [X]Self mutilation |
| ZX1M. | Shooting self |
| ZX1N. | Stabbing self |
| ZX1Q. | Throwing self in front of train |
| ZX1R. | Throwing self in front of vehicle |
| ZX1S. | Throwing self onto floor |

Table 2 Read codes used to identify eating disorders in GP data

| **Read Code** | **Description** |
| --- | --- |
| E271.00 | Anorexia nervosa |
| Eu50000 | [X]Anorexia nervosa |
| Eu50100 | [X]Atypical anorexia nervosa |
| E275100 | Bulimia (non-organic overeating) |
| Eu50200 | [X]Bulimia nervosa |
| Eu50211 | [X]Bulimia NOS |
| Eu50212 | [X]Hyperorexia nervosa |
| Eu50300 | [X]Atypical bulimia nervosa |
| Eu50400 | [X]Overeating associated with other psychological disturbncs |
| Eu50411 | [X]Psychogenic overeating |
| E275.00 | Other and unspecified non-organic eating disorders |
| E275000 | Unspecified non-organic eating disorder |
| E275200 | Pica |
| E275y00 | Other specified non-organic eating disorder |
| E275z00 | Non-organic eating disorder NOS |
| Eu50.00 | [X]Eating disorders |
| Eu50y00 | [X]Other eating disorders |
| Eu50y11 | [X]Pica in adults |
| Eu50y12 | [X]Psychogenic loss of appetite |
| Eu50z00 | [X]Eating disorder, unspecified |
