# Supplementary material for: Clinical management and mortality risk in those with eating disorders and self-harm: e-cohort study using the SAIL databank
Source: BJPsych Open. 2021 Mar 19;7(2):e67. doi: 10.1192/bjo.2021.23 (PMC8058850; doi:10.1192/bjo.2021.23)
Supplement: Supplementary file 1 [file bjosup.zip › S2056472421000235sup003.zip!/Supplementary_Table_2.docx]

**Supplementary Table 2.** Adjusted and unadjusted HRs (95% CI) by diagnosis

|  | Adjusted HR(95% CI) | Unadjusted HR(95% CI) |
| --- | --- | --- |
| No diagnosis | Reference | Reference |
| Self-harm only | 5.3(5.2-5.5) | 4.0(3.8-4.1) |
| Eating disorder only | 4.1(3.4-4.9) | 1.5(1.3-1.8) |
| Self-harm and eating disorders | 6.8(5.4-8.6) | 2.3(1.8-2.9) |
