# Supplementary material for: Clinical management and mortality risk in those with eating disorders and self-harm: e-cohort study using the SAIL databank
Source: BJPsych Open. 2021 Mar 19;7(2):e67. doi: 10.1192/bjo.2021.23 (PMC8058850; doi:10.1192/bjo.2021.23)
Supplement: Supplementary file 1 [file bjosup.zip › S2056472421000235sup003.zip!/Supplementary_Table_1.docx]

**Supplementary Table 1.** SMR, average YLL^a^ and average age at death by cause of death and diagnosis.

|  | | SMR | YLL | Average age at death |
| --- | --- | --- | --- | --- |
| All Cause | Self-harm only | 3.2(3.1-3.3) | 27.7(13.4) | 47(13.4) |
|  | Eating disorders only | 1.2(1.0-1.4) | 28.3(14.1) | 46(14.1) |
|  | Self-harm and eating disorders | 1.9(1.5-2.4) | 34.6(11.9) | 40(12.0) |
|  | Total population |  | 14.9(12.1) | 60(12.2) |
| Natural | Self-harm only | 2.1(2.0-2.2) | 23.2(12.0) | 51(12.0) |
|  | Eating disorders only | 1.2(1.0-1.4) | 27.7(14.3) | 47(14.3) |
|  | Self-harm and eating disorders | 1.1(0.8-1.5) | 30.4(12.0) | 44(11.9) |
|  | Total population |  | 13.7(10.7) | 61(10.8) |
| Unnatural | Self-harm only | 17.1(16.3-17.9) | 34.7(12.4) | 40(12.4) |
|  | Eating disorders only | 1.7(0.9-2.8) | 33.2(11.3) | 41(11.3) |
|  | Self-harm and eating disorders | 11.5(7.9-16.3) | 39.7(9.7) | 35(9.7) |
|  | Total population |  | 31.5(15.6) | 43(15.7) |
| Suicide | Self-harm only | 23.4(22.0-24.9) | 34.2(12.8) | 40(12.8) |
|  | Eating disorders only | 1.3(0.4-3.1) | 28.3(11.4) | 46(11.4) |
|  | Self-harm and eating disorders | 15.9(9.6-24.9) | 41.5(8.1) | 33(8.1) |
|  | Total population |  | 32.1(14.2) | 42(14.2) |
| a. Average YLL calculated to age 75 | | | | |
